# Quorum Quenching Lactonase Strengthens Bacteriophage and Antibiotic Arsenal Against *Pseudomonas aeruginosa* Clinical Isolates

**DOI:** 10.3389/fmicb.2019.02049

**Published:** 2019-09-03

**Authors:** Sonia Mion, Benjamin Rémy, Laure Plener, Fabienne Brégeon, Eric Chabrière, David Daudé

**Affiliations:** ^1^Aix-Marseille University, IRD, APHM, MEPHI, IHU-Méditerranée Infection, Marseille, France; ^2^Gene&GreenTK, Marseille, France; ^3^Service des Explorations Fonctionnelles Respiratoires Centre Hospitalo Universitaire Nord, Pôle Cardio-Vasculaire et Thoracique, Assistance Publique des Hôpitaux de Marseille, Marseille, France

**Keywords:** quorum quenching, quorum sensing, lactonase, AHL, *Pseudomonas aeruginosa*, multidrug resistant bacteria, antibiotics, bacteriophages

## Abstract

Many bacteria use quorum sensing (QS), a bacterial communication system based on the diffusion and perception of small signaling molecules, to synchronize their behavior in a cell-density dependent manner. QS regulates the expression of many genes associated with virulence factor production and biofilm formation. This latter is known to be involved in antibiotic and phage resistance mechanisms. Therefore, disrupting QS, a strategy known as quorum quenching (QQ), appears to be an interesting way to reduce bacterial virulence and increase antibiotic and phage treatment efficiency. In this study, the ability of the QQ enzyme *Sso*Pox-W263I, a lactonase able to degrade acyl-homoserine lactones, was investigated for quenching both virulence and biofilm formation in clinical isolates of *Pseudomonas aeruginosa* from diabetic foot ulcers, as well as in the PA14 model strain. These strains were further evolved to resist to bacteriophage cocktails. Overall, 10 antibiotics or bacteriophage resistant strains were evaluated and *Sso*Pox-W263I was shown to decrease pyocyanin, protease and elastase production in all strains. Furthermore, a reduction of more than 70% of biofilm formation was achieved in six out of ten strains. This anti-virulence potential was confirmed *in vivo* using an amoeba infection model, showing enhanced susceptibility toward amoeba of nine out of ten *P. aeruginosa* isolates upon QQ. This amoeba model was further used to demonstrate the ability of *Sso*Pox-W263I to enhance the susceptibility of sensitive and phage resistant bacteria to bacteriophage and antibiotic.

## Introduction

*Pseudomonas aeruginosa* is an opportunistic human pathogen involved in numerous diseases from otitis to keratitis, wound and burn infections, pneumonia and urinary tract infections (Driscoll et al., 2007). *P. aeruginosa* isolates are among the most frequently found antibiotic-resistant pathogens involved in diabetic foot infections, their presence is usually associated with morbidity (Ertugrul et al., 2012). Surveillance of *P. aeruginosa* infections has revealed trends of increasing resistance to antibiotic treatments (Breidenstein et al., 2011) and the priority to support research and development of effective drugs against antibiotic-resistant *P. aeruginosa* was recently defined as critical by the World Health Organization (WHO) (Tacconelli et al., 2018).

This last decade, bacteriophage (phage) therapy has regained interest as a new weapon to treat antibiotic-resistant infections (Rolain et al., 2015; Domingo-Calap et al., 2016) and is under consideration to treat *P. aeruginosa* infections. Phages are the most abundant predator of bacteria in nature (Suttle, 2005) and have been domesticated, especially in Eastern Europe, to treat enteric infections, such as cystic fibrosis (Rolain et al., 2015), dysentery (Salmond and Fineran, 2015), diabetic foot infection, chronic osteomyelitis and other surgical and wound infections (Kutter et al., 2010). Phages offer the advantage of specifically targeting their host bacteria by being harmless to the commensal flora (Loc-Carrillo and Abedon, 2011) and human cells (Domingo-Calap et al., 2016). In western countries, clinical trials have been conducted to assay the therapeutic potential of phages (Rhoads et al., 2009; Wright et al., 2009; Kakasis and Panitsa, 2018), for example to treat *P. aeruginosa* infected burns (Jault et al., 2018). However, as for antibiotics, phages suffer resistance phenomena that may hinder the development of bacteriophage-based therapy (Jault et al., 2018). Finding new therapeutic strategies to limit bacterial resistance is thus of great interest.

In this way, another alternative to treat *P. aeruginosa* infections aims to highjack its communication system referred to as quorum sensing (QS). In *P. aeruginosa*, QS mainly relies on the secretion and perception of *N*-acyl homoserine lactones (AHL) to orchestrate its behavior, including virulence and biofilm formation as well as the CRISPR-Cas defense system, in a cell-density dependent manner (Bassler and Losick, 2006; Høyland-Kroghsbo et al., 2017). Interconnections between bacterial QS and susceptibility to bacteriophage infections were also identified (Qin et al., 2017; Saucedo-Mora et al., 2017). Disrupting QS, a strategy referred to as quorum quenching (QQ), is highly attractive to counteract bacterial virulence by using QS inhibitors (QSI) or QQ enzymes (QQE). Among QQE, special attention has been paid to the robust lactonase *Sso*Pox-W263I (Hiblot et al., 2013) that was proved to efficiently inhibit virulence *in vitro* in model and clinical strains of *P. aeruginosa* (Guendouze et al., 2017) and to drastically decrease mortality in a rat pulmonary infection model (Hraiech et al., 2014). Furthermore, the impact of *Sso*Pox-W263I on CRISPR-Cas gene expression of *P. aeruginosa* was recently demonstrated in both model and clinical strains suggesting that enzymatic QQ may modulate bacterial susceptibility to bacteriophages (Mion et al., 2019).

Here, we consider the use of *Sso*Pox-W263I in clinical isolates of *P. aeruginosa* from diabetic foot ulcers together with the model strain PA14 and further evolved these strains to resist bacteriophages. Knowing that the evolutionary selection of phage resistance in bacteria can induce phenotypic shifts (Labrie et al., 2010), we focused our interest on determining the potential of QQ to control antibiotic and phage-resistant bacteria. Our results show that *Sso*Pox-W263I is efficient to decrease virulence or biofilm in these multi-resistant strains, both *in vitro* and *in vivo* using an amoeba model. In the last part, we investigate the QQE treatment to enhance the therapeutic effect of antibiotic and phage when used as a co-treatment in the amoeba infection model. This study shows that QQ is an interesting strategy to treat bacterial infections that can be used to strengthen the effect of antimicrobial treatments.

## Materials and Methods

### Bacterial Strains and Growth Conditions

Experiments were conducted using model strain PA14 and three clinical isolates of *P. aeruginosa*, isolated from diabetic patients of the Nimes University Hospital presenting diabetic foot infections. All the patients received an oral information, were anonymized and gave a non-opposition statement to bacterial storage. This study was approved by the local ethics committee (South Mediterranean III) and was carried out in accordance with the Declaration of Helsinki as revised in 2008. Clinical isolates of *P. aeruginosa* and model strain PA14 (UCBPP-PA14) were inoculated from a single colony and pre-cultivated during 6 h at 37°C in Luria Bertani (LB) medium (10 g l^–1^ NaCl, 10 g l^–1^ Tryptone, 5 g l^–1^ yeast extract) with agitation at 650 rpm. Then, precultures were diluted by a 1,000 factor in MOPS minimal medium complemented with nitrogen (15 mM NH_4_Cl), iron (5 μM Fe_2_SO_4_), phosphate (4 mM K_2_HPO_4_) and glutamate (25 mM) as carbon source (MOPS glutamate) (Welsh and Blackwell, 2016) and cultures were incubated at 37°C under agitation at 650 rpm. Enzymes were added, when indicated, at 0.5 mg ml^–1^. For virulence factor and biofilm formation analysis cultures were incubated during 20 h.

### Phage Production and Isolation

A bacteriophage cocktail (Intesti-bacteriophage, Microgen, Russia) was used for this study. Isolated phage ΦIntesti-PA14 corresponds to the isolation and concentration of plaque-forming unit (PFU) formed by the phage cocktail on *P. aeruginosa* PA14 according to the following protocol.

The double agar overlay plaque assay was used to determine the phage titer and isolate phages from phage cocktail (Kropinski et al., 2009). 500 μl of an overnight culture of bacteria was added to 4.5 ml of molten soft LB-agar (0.75%) and overlaid onto a hard LB-agar plate. Once dry, 10 μl drops of phage cocktail were spotted on the soft LB-agar layer. Plates were incubated overnight at 37°C. Lytic plaques were collected and suspended in 500 μl of MgSO_4_ (10 mM). After chloroform treatment and 10 min of centrifugation at 4,500 *g*, the supernatant was filtered at 0.22 μm. The resulting phage suspension was again spotted on a double-layer plate, and the experiment was repeated until a 10^8^ PFU ml^–1^ suspension was obtained. To estimate the phage titer, serial dilutions (from 10^0^ to 10^–10^) of phage suspension were performed in MgSO_4_ (10 mM), then 10 μl of each dilution was spotted on the double-layer plate. The plate was incubated overnight at 37°C. The titer (PFU ml^–1^) was determined by the calculation of lytic PFU for 1 ml of phage suspension.

### Isolation of Phage Resistant *P. aeruginosa* Bacteria

Precultures were performed by inoculating one colony of the initial strains in LB medium during 6–8 h, then the precultures were diluted by a 10,000 factor in MOPS glutamate, 3 ml were transferred in each well of a 12-well plate. 10, 50, or 100 μl of bacteriophage containing cocktail (Intesti-bacteriophage, Ìicrogen, Russia) were added to each well. The plate was incubated at 37°C overnight under agitation at 650 rpm. The OD 600 nm was then measured using a plate reader (Synergy HT, BioTek). Cultures showing complete lysis were diluted in a solution of phosphate saline buffer (PBS) and 20% glycerol. The dilutions were then plated on LB agar. Plates were incubated at 37°C overnight.

After 16 h of incubation, the isolated colonies were picked, cultivated in LB medium and stocked in 20% glycerol at −150°C. The tolerance of the isolated bacteria to the phage cocktail was verified by exposing the strains to the same conditions as those used for their isolation and by verifying that the final OD 600 nm was indeed higher for the resistant strain than for the parental strain.

### Protein Production and Purification

Two *Sso*Pox variants namely W263I and 5A8 were used as QQ and inactive enzymes, respectively. Productions were realized as previously described (Hiblot et al., 2012, 2013; Hraiech et al., 2014). Briefly, *Escherichia coli* BL21 (DE3)-pGro7/GroEL cells (TaKaRa), carrying plasmid pET22b-*Sso*Pox-W263I or pET22b-*Sso*Pox-5A8, were cultivated in ZYP-5052 medium complemented with 100 μg ml^–1^ ampicillin and 34 μg ml^–1^ chloramphenicol at 37°C until OD 600 nm reached 0.8–1. The expression of chaperone proteins was induced by adding L-arabinose at a final concentration of 0.2% (w/v). At the same time, the temperature was reduced to 23°C and 0.2 mM of CoCl_2_ was added. After 20 h of incubation, the cells were harvested by centrifugation (4,400 *g*, 4°C, 20 min), the pellet was resuspended in lysis buffer [50 mM HEPES pH 8, 150 mM NaCl, 0.25 mg ml^–1^ lysozyme, 0.1 mM Phenylmethylsufonyl fluoride (PMSF) and 10 mg ml^–1^ DNaseI] and stored at −80°C during 16 h. Frozen cells were thawed at 37°C during 15 min and lysed by three steps of 30 s sonication (QSonica sonicator Q700, amplitude at 45). Cell debris were removed by centrifugation (21,000 *g*, 4°C, 15 min). Crude extract was incubated during 30 min at 80°C and then centrifuged to precipitate *E. coli* proteins (21,000 *g*, 4°C, 30 min). The enzyme was then concentrated by overnight incubation at 4°C in 75% ammonium sulfate. After resuspension in activity buffer (50 mM HEPES pH 8, 150 mM NaCl, 20.2 mM CoCl_2_) ammonium sulfate was eliminated by desalting (HiPrep 26/10 desalting, GE Healthcare, ÄKTA Avant). The protein sample obtained was concentrated to 2 ml and then loaded on a size-exclusion chromatography column and purified to homogeneity (HiLoad 16/600 Superdex^TM^ 75pg, GE Healthcare, ÄKTA Avant). Protein purity was checked by migration on 10% SDS-PAGE and protein concentration was measured using a spectrophotometer NanoDrop 2000 (Thermo Scientific).

### Antibiograms

Antibiotic sensitivity of the strains was determined on a Mueller Hinton agar (BioMerieux). The disk diffusion method was realized using the following antibiotics: amikacin (30 μg), cefepime (30 μg), ceftazidime (30 μg), ciprofloxacin (5 μg), doxycycline (30 μg), fosfomycin (50 μg), imipenem (10 μg), nitrofurantoin (300 μg), piperacillin/tazobactam (85 μg), ticarcillin (75 μg), ticarcillin/clavulanate (85 μg), trimethoprim/sulfamethoxazole (25 μg), tobramycin (10 μg), rifampicin (30 μg). The results were interpreted according to the EUCAST guidelines (European Society of Clinical Microbiology and Infectious Diseases, 2018^1^) using the Scan^®^ 1200 (Interscience) (Diop et al., 2016).

### Analysis of Virulence Factor Production

Virulence factor productions for the different strains were determined *in vitro* after a 20-h culture in presence of 0.5 mg ml^–1^ *Sso*Pox-W263I or inactive mutant *Sso*Pox-5A8 as control.

#### Pyocyanin Production

Cell-free culture supernatants were prepared by centrifugation for 5 min at 12,000 *g*. Pyocyanin was extracted by mixing 500 μl of cell-free supernatant with 250 μl of chloroform (Price-Whelan et al., 2007). After vortexing and 5 min of centrifugation at 12,000 *g*, 200 μl of the bottom chloroform phase were transferred into a quartz 96-well plate. The absorbance was measured at 690 nm using a plate reader (Synergy HT, BioTek).

#### Proteolytic Activity

The protease activity was measured using azocasein (Sigma) (Chessa et al., 2000). 675 μl of PBS solution pH 7 were mixed with 50 μl of azocasein (30 mg ml^–1^ in water) and 25 μl of cell-free supernatant. After 2 h of incubation at 37°C, 125 μl of 20% (w/v) trichloroacetic acid were added to stop the reaction. The solution was then centrifugated for 10 min at 10,000 *g* and the absorbance of 200 μl of the supernatant was measured at 366 nm using a plate reader (Synergy HT, BioTek).

#### Elastolytic Activity

Elastase B activity was measured using elastin-Congo red conjugate (Sigma) degradation assay (Smith et al., 2003). 50 μl of cell-free supernatant were added to 150 μl of elastin-Congo red solution (5 mg ml^–1^ in 10 mM Tris–HCl and 1 mM CaCl_2_ buffer at pH 7.2) into a 96-well plate. The plate was then sealed with an aluminum membrane and incubated during 24 h at 37°C under agitation (300 rpm). After sedimentation of undigested elastin-Congo red conjugate 100 μl of the upper phase was transferred to an empty well and the absorbance was measured at 490 nm using a plate reader (Synergy HT, BioTek).

### Biofilm Formation

The biofilm formed in each well was quantified using crystal violet (Sigma) staining as previously described (Stepanović et al., 2000). Briefly, planktonic cells (non-attached) were first removed by washing the wells with 4 ml of PBS. The plates were then dried at 37°C and the biofilm was stained by adding 4 ml of crystal violet 0.05%, incubated for 3 min under agitation at 150 rpm. Then, the crystal violet was removed and each well was rinsed with 4 ml of PBS. Crystal violet was then resolubilized by adding 3 ml of ethanol 96%. 200 μl of the solution was transferred to a 96-well plate and the final concentration of crystal violet was measured at OD 595 nm using a plate reader (Synergy HT, BioTek).

### Virulence Assay Toward Amoeba

*In vivo* virulence assay was adapted from a previously described procedure using *P. aeruginosa* and *Acanthamoeba polyphaga Linc AP1* (Fenner et al., 2006). Briefly, 3 ml of bacterial culture were pelleted down and resuspended in Page’s amoeba saline (PAS) buffer (2 mM NaCl, 16 μM MgSO_4_, 27 μM CaCl_2_, 0.53 mM Na_2_HPO_4_, 1 mM KH_2_PO_4_, pH 6.9) after a culture in 6-well plates (Nunc^TM^, Thermo Scientific). *A. polyphaga Linc AP1* was cultivated during 2–3 days into peptone yeast extract glucose (20 g l^–1^ proteose peptone, 2 g l^–1^ yeast extract, 0.1 M glucose, 4 mM MgSO_4_, 0.53 mM CaCl_2_, 3.4 mM sodium citrate, 50 μM (NH_4_)_2_Fe(SO_4_)_2_, 2.5 mM KH_2_PO_4_, 1.3 mM Na_2_HPO_4_, pH 6.8) medium at 28°C (Fenner et al., 2006). Amoeba cells were recovered after centrifugation at 750 *g* and resuspended into PAS buffer to 10^5^ cells μl^–1^. Then, 1 ml of bacterial suspension was spread on a PAS agar plate and was left to dry at room temperature. At the center of each plate, 5 μl of *A. polyphaga* were spotted and dried at room temperature. Then, plates were incubated at 30°C over 7 days and amoeba propagation was followed by directly measuring the central spot with a ruler.

To test the combinatory effect of enzymatic and antibiotic treatments, the MOPS bacterial culture was treated with 10 μg ml^–1^ of enzyme (either *Sso*Pox-W263I or inactive variant *Sso*Pox-5A8 as control) and 25 μg ml^–1^ ciprofloxacin was added to PAS buffer during the resuspension step.

To test the combinatory effect of enzymatic and phage treatments, the MOPS bacterial culture was treated with 10 μg ml^–1^ of enzyme (either *Sso*Pox-W263I or inactive variant *Sso*Pox-5A8 as control) and 10^7^ PFU ml^–1^ of ΦIntesti-PA14 phage was added to PAS buffer during the resuspension step.

## Results

### Evaluating Sensitivity of Clinical Isolates to Antibiotics and Bacteriophage Cocktail

**FIGURE 1:**
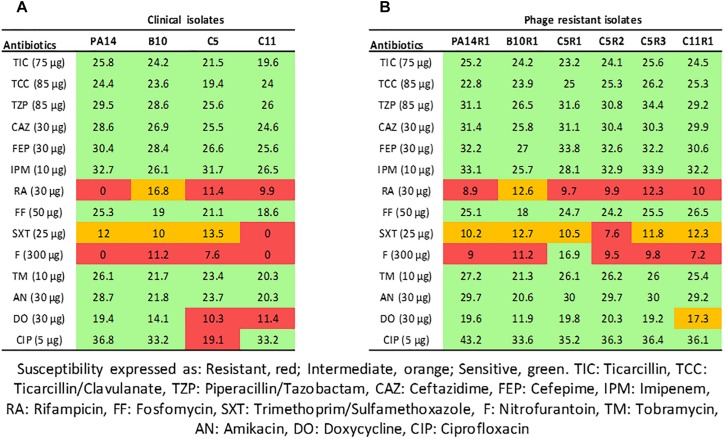
Interpretative zone diameters (mm) of 14 antibiotics used on clinical isolates of *Pseudomonas aeruginosa* **(A)** and their associated phage-resistant mutants **(B)**. Susceptibility is expressed as Resistant (red), Intermediate (orange), Sensitive (green).

PA14 is a model strain originally isolated from a burn wound (Soyza et al., 2013) and B10, C5, and C11 were isolated from diabetic foot ulcerations (Guendouze et al., 2017). Antibiotic susceptibility of the strains was evaluated using the disk diffusion method with 14 antibiotics and analyzed according the EUCAST recommendations (Figure 1A). The strains were non-susceptible (resistant or intermediate) to at least two different antibiotics tested belonging to rifamycin, sulfonamide or nitrofuran classes. All strains were found sensitive to the tested agents in β-lactam, aminoglycosides and fosfomycin antimicrobial classes which are commonly used to fight pseudomonal infections. In addition to antibiotic sensitivity, the impact of a commercial bacteriophage cocktail on the strains was evaluated. Interestingly, all the strains were sensitive to the cocktail resulting in drastic decreases in cell density (Supplementary Figure 1). These results confirm that bacteriophage-based therapy may constitute an alternative to antibiotherapies in case of resistant infections.

### Isolation and Characterization of Phage-Resistant Variants

**FIGURE 2:**
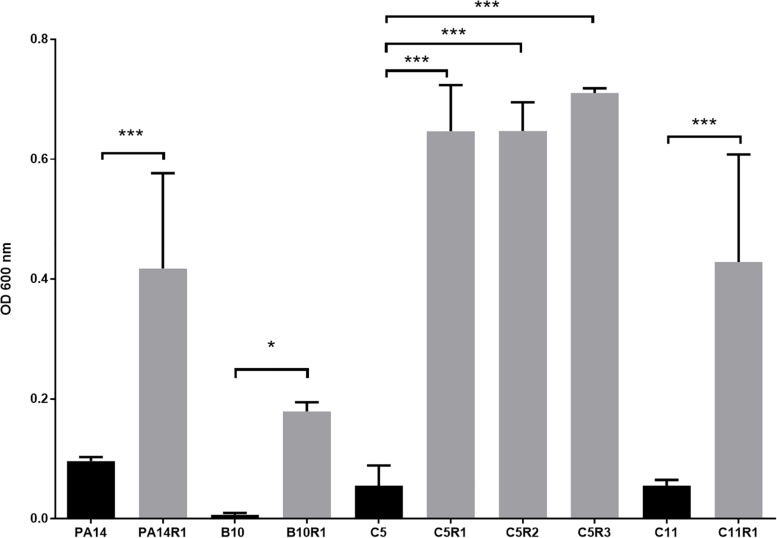
Growth of phage resistant (gray) and the associated parental strains (black) in presence of phage cocktail. For each strain, bars represent the mean density (OD 600 nm) after 16 h of incubation in MOPS glutamate with phage cocktail. Error bars represent the standard deviations of three replicated experiments. ^∗^*p*-values < 0.05; ^∗∗∗^*p*-values < 0.001 according to Student’s *t*-test or ANOVA analysis.

Although bacteriophages were virulent to all four strains, resistance phenomena were rapidly observed after exposure of PA14, B10, C5, and C11 to three different concentrations of phage cocktail for 16 h. For PA14, B10, and C11 one mutant was isolated from each strain: PA14R1, B10R1, and C11R1, respectively. Three different mutants, presenting different phenotypes, were isolated from C5: C5R1, C5R2, and C5R3. The newly isolated mutants were cultured in the presence of the same amount of phage cocktail as that used for their isolation to confirm their resistance (i.e., 100 μl for PA14 and PA14R1, 50 μl for B10 and B10R1 and 10 μl for C5, C5R1, C5R2, C5R3, C11, and C11R1). Cell density was compared to the initial strains in the presence of phages after a 16-h culture by measuring the OD 600 nm (Figure 2). In the presence of phages, growth was 4 to 7 times higher for all mutants than for parental strains (Figure 2). The resistance of the isolated mutants against the phage cocktail was thereby clearly highlighted (Figure 2). Antibiotic sensitivity patterns of the phage-resistant strains were further evaluated (Figure 1B). As for parental isolates, phage resistant strains were non-susceptible (resistant or intermediate) to at least two different antibiotics tested belonging to rifamycin, sulfonamide or nitrofuran classes. Phage-resistant strains were found to be sensitive to the tested agents in β-lactam, aminoglycosides, cephalosporins, carbapenems and fosfomycin antimicrobial classes. As noticed for B10, B10R1 showed intermediate resistance to rifampicin while all other strains were resistant to this antibiotic. Interestingly, the acquisition of bacteriophage resistance was detrimental to antibiotic resistance in the resistant clones isolated from C5. C5R1, C5R2, and C5R3 lost their resistance against doxycycline and ciprofloxacin, C5R1 being also sensitive to nitrofurantoin conversely to C5, C5R2, or C5R3. Similarly, C11R1 exhibited a lower tolerance to trimethoprim/sulfamethoxazole and doxycycline than C11.

### Quenching Virulence Factors of Antibiotic or Phage Resistant Clones *in vitro*

**FIGURE 3:**
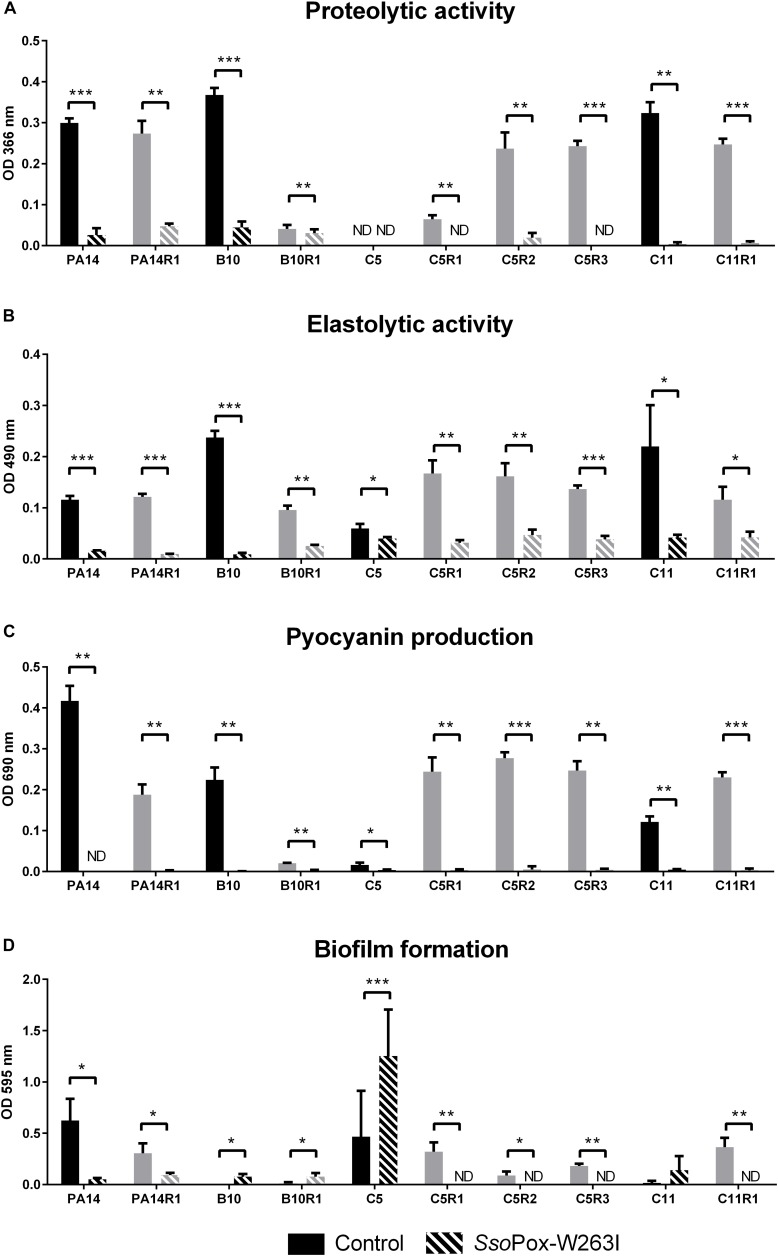
Effect of *Sso*Pox-W263I on virulence factors of phage resistant mutants (gray) and the associated parental strains (black) *in vitro*. For each strain, bars represent the mean of protease **(A)**, elastase **(B)**, pyocyanin **(C)** and biofilm **(D)** levels of three experiments for treated culture with 0.5 mg ml**^–^**^1^ *Sso*Pox-W263I (striped bar) or inactive mutant 5A8 as negative control (empty bar). ND: not detected. Error bars represent the standard deviations of three experiments. ^∗^*p*-values < 0.05; ^∗∗^*p*-values < 0.01; ^∗∗∗^*p*-values < 0.001 according to Student’s *t*-test.

The QQ effect of *Sso*Pox-W263I on *P. aeruginosa* isolates PA14, C5, C11, B10 and their bacteriophage resistant counterparts was investigated by measuring the production of three typical virulence factors *in vitro*: pyocyanin, protease, elastase as well as biofilm production. Under the tested conditions, the addition of *Sso*Pox-W263I significantly reduced the three virulence factors for all strains compared to controls (Figures 3A–C). All tested strains produced detectable levels of pyocyanin and elastolytic activity. Pyocyanin levels were reduced by more than 75% in all strains upon enzymatic treatment, while elastase levels were reduced by at least 60% in nine out of ten strains with the addition of *Sso*Pox-W263I (C5 elastase level being reduced by only 25%). Proteolytic activity was detectable for all tested strains but one: the parental isolate C5 which did not produce sufficient levels of proteases with or without *Sso*Pox-W263I treatment to be detected. Conversely, the three phage-resistant derivatives obtained from C5 had each detectable protease activity in the absence of enzyme and this activity was completely extinguished by the addition of *Sso*Pox-W263I (Figure 3A). Biofilm production was significantly reduced by more than 70% in six strains when treated with *Sso*Pox-W263I (Figure 3D). Interestingly, the production of these four factors differed between the parental strains and their phage-resistant derivatives (Figure 3). It appears that the selection for phage-resistant clones did not result in the selection for mutants with only increased or only decreased biofilm formation. Similarly phage-resistant mutants did not all increase nor decrease virulence factor secretion as compared to their parental strains. Thereby no common trade-off due to the selection of phage resistant bacteria can be drawn at the level of these four phenotypic traits. Altogether, these results show the efficiency of *Sso*Pox-W263I in reducing the amount of three virulence factors characteristic of *P. aeruginosa* and modulating the production of biofilm *in vitro* in both antibiotic and phage-resistant isolates.

### Evaluation of Quorum Quenching in Amoeba, Virulence Model

To further confirm the potential of *Sso*Pox-W263I to decrease the virulence of bacterial isolates, the *in vivo* protecting effect of the enzyme was assayed using the amoeba *A. polyphaga*. The virulence of treated and control bacteria toward amoeba was assayed by measuring the propagation of *A. polyphaga* on a plate flooded by a pretreated bacterial lawn (Fenner et al., 2006).

**FIGURE 4:**
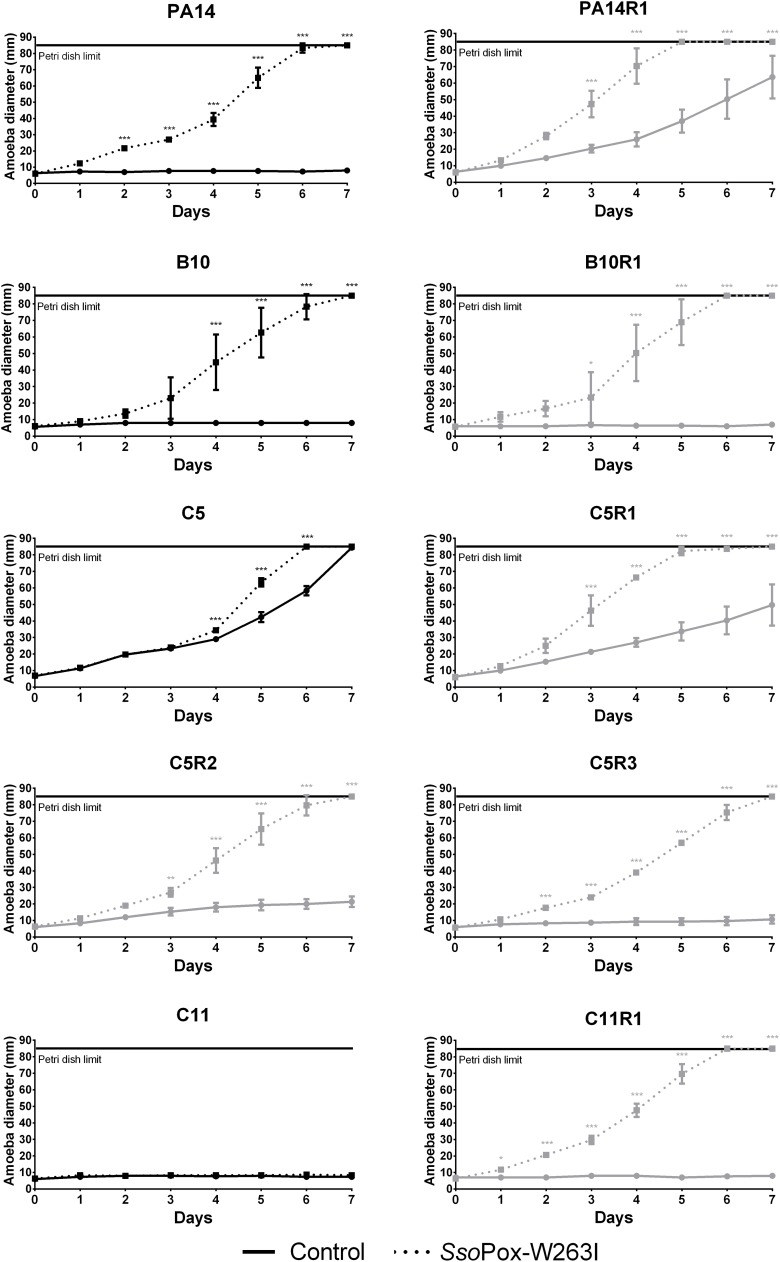
Effect of *Sso*Pox-W263I on the virulence of clinical isolates (black) and phage resistant mutants (gray) against *A. polyphaga Linc AP1*. For each strain, curves represent the mean diameter of amoeba at different days of three experiments after incubation in presence of bacteria treated by 0.5 mg ml^–1^ of mutant *Sso*Pox-W263I (dotted line) or inactive mutant 5A8 (full line) as negative control. Error bars represent the standard deviations of three experiments. ^∗^*p*-values < 0.05; ^∗∗^*p*-values < 0.01; ^∗∗∗^*p*-values < 0.001 according to ANOVA analysis.

Overall, *Sso*Pox-W263I treatment decreased the virulence toward *A. polyphaga* of 9 out of 10 strains. No effect of QQ was observed for C11, for which virulence toward *A. polyphaga* remained unchanged with or without *Sso*Pox-W263I treatment, but its phage resisting mutant C11R1 recovered a high sensitivity to the amoeba upon treatment (Figure 4). C5, C5R1, and PA14R1 were initially not virulent enough to prevent the propagation of amoeba in the control condition; however, treatment by *Sso*Pox-W263I significantly enhanced its expansion (Figure 4). Consistently with *in vitro* observations on virulence factor production, the results obtained *in vivo* confirmed that parental and resistant strains behave differently, especially for PA14 and C5 (Figure 4). *Sso*Pox-W263I treatment showed a benefic effect in all but one case and no negative effects were observed, highlighting the efficiency of the QQ treatment in reducing the virulence of antibiotic and phage resistant isolates in an *in vivo* model.

### Combined Effect of Enzymatic and Antimicrobial Treatment

**FIGURE 5:**
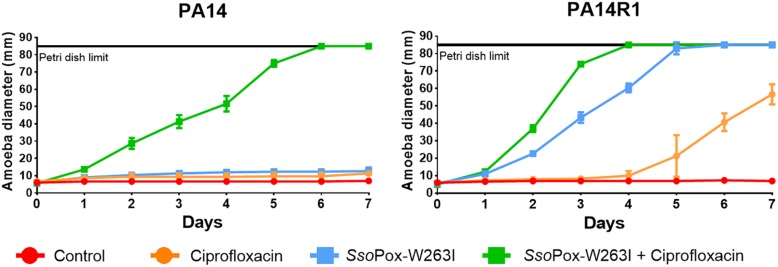
Combinatory effect of *Sso*Pox-W263I and ciprofloxacin on the virulence of clinical isolates and phage resistant mutants against *A. polyphaga Linc AP1*. For each strain, curves represent the mean diameter of amoeba at different days after incubation in presence of bacteria without treatment (red) or treated with 25 mg ml**^–^**^1^ ciprofloxacin (orange), 10 μg ml**^–^**^1^ *Sso*Pox-W263I (blue), or 10 μg ml**^–^**^1^ *Sso*Pox-W263I and 25 mg ml**^–^**^1^ ciprofloxacin (green). Error bars represent the standard deviations of three experiments.

**FIGURE 6:**
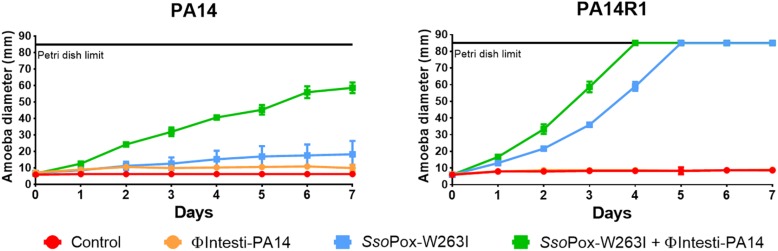
Combinatory effect of *Sso*Pox-W263I and isolated phage on the virulence of PA14 and its phage resistant mutant against *A. polyphaga Linc AP1*. For each strain, curves represent the mean diameter of amoeba at different days after incubation in presence of bacteria without treatment (red) or treated with 10^7^ PFU ml^–1^ ΦIntesti-PA14 (orange), 10 μg ml^–1^ *Sso*Pox-W263I (blue), or 10 μg ml^–1^ *Sso*Pox-W263I and 10^7^ PFU ml^–1^ ΦIntesti-PA14 (green). Error bars represent the standard deviations of three experiments.

Considering that PA14 displayed antibiogram comparable to most clinical isolates tested in this study, PA14 and its phage resistant mutant PA14R1 were further used as representative candidates to evaluate the combined effect of *Sso*Pox-W263I and antibiotic treatment using the amoeba infection model (Figure 5), then the combined effect of *Sso*Pox-W263I and phage treatment was also assayed (Figure 6). As observed in our first experiment, with an enzyme concentration of 500 μg ml^–1^, QQ increases the sensitivity toward amoeba. Hence, to better assay the combined effect of enzymatic and antimicrobial treatments, lower doses of *Sso*Pox-W263I were considered. Thus, following a dose response experiment on PA14, 10 μg ml^–1^ of *Sso*Pox-W263I was used with antimicrobial as it had a lower impact on the virulence (Supplementary Figure 2).

Synergistic effect was observed with a treatment of *Sso*Pox-W263I and 25 mg ml^–1^ ciprofloxacin on PA14, PA14R1 (Figure 5). At these concentrations, each treatment alone had no effect on the virulence of PA14 against the amoeba and the amoeba could not grow. However, with the combined treatment (ciprofloxacin + *Sso*Pox-W263I), the amoeba was able to completely colonize the Petri dish in 6 and 7 days, respectively, for PA14. PA14R1 virulence was slightly impacted by the antibiotic treatment alone or the QQE treatment alone, but the highest effect was observed with the combined treatment: the Petri dish limit (8.5 cm) was reached 1 day earlier than with the QQE treatment alone, and the growth of amoeba started after 1 day with the combined treatment against 4 days with ciprofloxacin alone.

As the composition of the commercial cocktail was toxic for amoeba, PA14 lytic phages were purified from the cocktail and concentrated to 10^8^ PFU ml^–1^. To test the combined effect of *Sso*Pox-W263I and phages, ΦIntesti-PA14 was used alone or in combination with the enzyme against PA14 and PA14R1. The isolated phages did not impact the growth of the amoeba. For both PA14 and PA14R1, amoeba growth was faster with *Sso*Pox-W263I alone, yet the combined treatment led to a further increase of amoeba growth (Figure 6).

## Discussion

In this study, four strains of *P. aeruginosa* were used, including three clinical isolates from diabetic foot infections. The antibiotic resistance profiles of the strains revealed that all the clinical isolates presented a significant tolerance to rifampicin, trimethoprim/sulfamethoxazole and nitrofurantoin, confirming previous observations regarding the increasing rate of multi-resistance in diabetic foot infections in the recent years (Lipsky, 2016).

To address antibioresistance issues, bacteriophages and QQ have emerged as promising therapeutic approaches. As with antibiotics, the use of bacteriophages suffers from rapid resistance phenomena such as the formation of a biofilm, the modification of phage receptor expression that can reduce phage entry (Chapman-McQuiston and Wu, 2008), or the cell adaptive inducible CRISPR-Cas (clustered regularly interspaced short palindromic repeat and CRISPR associated proteins) system that recognizes and degrades phage DNA (Barrangou et al., 2007). Interestingly, it has been extensively demonstrated that QQ reduces biofilm formation in *P. aeruginosa*, thereby increasing antimicrobial treatment efficacy, and a recent study has underlined that QS disruption can also decrease phage resistance by inhibiting QS stimulation of the CRISPR-Cas system (Høyland-Kroghsbo et al., 2017). The efficacy of *Sso*Pox-W263I to modulate CRISPR-Cas system regulation of proteobacteria, including *P. aeruginosa*, was recently demonstrated (Mion et al., 2019). In addition, recent studies have shown that even without involvement of CRISPR-Cas defense, the phage infection outcome could be different in QS-deficient mutant of *P. aeruginosa* than in wild type strains (Qin et al., 2017; Saucedo-Mora et al., 2017). As antibiotic or phage resistance can induce life-threatening complications, we evaluated the potential of QQ to act as an alternative therapeutic approach.

First, we selected six mutants derived from the four initial strains as phage resistant toward a commercial phage cocktail. The virulence profiles of each strain were then evaluated and the efficacy of three therapeutic approaches (antibiotics with ciprofloxacin, bacteriophages and QQ with *Sso*Pox-W263I) on the different strains was further assessed alone or in combinations.

In several cases bacteriophage resistance did not affect antibiotic resistance, although the selection pressure induced by the phage cocktail resulted in a loss of resistance to ciprofloxacin and doxycycline in the mutant strains isolated from C5. This evolutionary trade-off is coherent with a previous study, where the phage OMKO1 which targets OprM, the porin of a multi-drug efflux system of *P. aeruginosa* as receptor-binding site, selected resistant bacteria harboring a change in the efflux mechanism with increased sensitivity to different antibiotic classes, such as tetracycline and fluoroquinolone (Chan et al., 2016).

To determine the potential of QQ treatment on the different strains, four QS-regulated traits, biofilm formation and the production of three virulence factors (pyocyanin, protease and elastase) were measured *in vitro*. As reported in other studies, some phage resistant bacteria such as C5R1, C5R2, and C5R3 exhibited higher level of virulence factor production than the initial strain (Hosseinidoust et al., 2013). The ability of *Sso*Pox-W263I as QQ agent to decrease virulence in the different strains was also assayed. The QQ treatment significantly reduced the production of the virulence factors for the initial strains as well as their phage resistant mutants, showing that bacteria resistant to phage treatment conserved a functional QS system which can be inhibited by enzyme-mediated QQ. Strains harboring a higher virulence profile were also efficiently quenched using *Sso*Pox-W263I. In order to correlate *in vitro* production of virulence factors, biofilm formation and *in vivo* virulence, an amoeba-based assay was developed. Amoeba are eukaryotic organisms able to feed on bacteria. Their phagocytosis and digestion mechanisms are similar to those of macrophage bacterial elimination (Greub and Raoult, 2004), thus amoeba are frequently used to test *in vivo* virulence of bacteria (Rémy et al., 2018). The model of *A. polyphaga* feeding on *P. aeruginosa*, where the growth of the amoeba directly indicates the pathogenicity level of the bacteria, was chosen to assess the efficiency of QQ treatment with *Sso*Pox-W263I. The results showed that the inhibition of virulence by the QQ treatment was efficient for 9 out of 10 strains, allowing the growth of the amoeba, independently from the resistance profiles to antibiotics or bacteriophages. Interestingly, *P. aeruginosa* was previously proved to use type III secretion system (T3SS) to kill biofilm-associated amoebae potentially suggesting that differences observed upon enzymatic treatment could be related in the modification of T3SS regulation (Matz et al., 2008). Although variable enzyme effects on virulence factor production or biofilm formation were observed *in vitro* depending on the strains, our results indicate that QQ is efficient *in vivo* on most of the antibiotic or phage resistant clinical isolates. This highlights the potential of enzymatic QQ treatment as an interesting alternative in case of therapeutic dead ends.

Furthermore, we evaluated the potential of *Sso*Pox-W263I to act as a complement of antibiotics or bacteriophages to counteract *P. aeruginosa* virulence toward *A. polyphaga*. Using lower concentrations of *Sso*Pox-W263I, the combination of QQ and ciprofloxacin enhanced the growth of the amoeba for ciprofloxacin-sensitive strains PA14 and PA14R1 as compared to the antibiotic or the enzymatic treatment alone. Consistently with previous reports using the lactonase from *Bacillus* sp. ZA12 with ciprofloxacin in a murine burn infection model (Gupta et al., 2015), our results show that enzymatic QQ works in synergy with fluoroquinolones in sensitive strains decreasing efficiently the amount of antibiotics required to fight bacterial infections.

In addition to antibiotics, the synergy of *Sso*Pox-W263I with bacteriophages was underlined. Synergistic effects were observed when *P. aeruginosa* PA14 was treated by the combined actions of isolated phage ΦIntesti-PA14 and *Sso*Pox-W263I. Interestingly, the synergy was also observed on PA14R1 strain, which was resistant to the phage cocktail. In concordance with these observations, recent reports showed close relationships between QS and phage tolerance mechanisms in *P. aeruginosa* (Mion et al., 2018).

Altogether, the results obtained *in vitro* and *in vivo* show that *Sso*Pox-W263I is efficient to decrease bacterial virulence in model and clinical isolates of *P. aeruginosa* and constitute a proof of concept suggesting that enzymatic QQ can strengthen the therapeutic arsenal available against *P. aeruginosa* infections by enhancing the efficiency of available treatments including bacteriophages or antibiotics. In addition, it has been shown that this enzyme, issued from an extremophilic organism, resists harsh industrial conditions (Rémy et al., 2016) confirming its tremendous potential for biopharmaceutical applications and should now be evaluated on mammalian models in order to reach clinical trials.

## Data Availability

The datasets generated for this study are available on request to the corresponding author.

## Author Contributions

SM, BR, LP, FB, DD, and EC designed the study and wrote the manuscript. SM, BR, and LP performed the experiments. SM, BR, LP, and DD analyzed the data.

## Conflict of Interest Statement

EC has a patent WO2014167140 A1 licensed to Gene&GreenTK. BR, LP, DD, and EC report personal fees from Gene&GreenTK during the conduct of the study. The remaining authors declare that the research was conducted in the absence of any commercial or financial relationships that could be construed as a potential conflict of interest.

## Footnotes

1 http://www.eucast.org/clinical_breakpoints/
