# Supplementary material for: Quorum Quenching Lactonase Strengthens Bacteriophage and Antibiotic Arsenal Against Pseudomonas aeruginosa Clinical Isolates
Source: Front Microbiol. 2019 Sep 3;10:2049. doi: 10.3389/fmicb.2019.02049 (PMC6734170; doi:10.3389/fmicb.2019.02049)
Supplement: Supplementary file 1 [file Data_Sheet_1.docx]

## Supplementary information

# Quorum quenching lactonase strengthens bacteriophage and antibiotic arsenal against *Pseudomonas aeruginosa* resistant isolates

Authors: S. Mion^1^, B. Rémy^1,2^, L. Plener^2^, F. Brégeon^1,3^, E. Chabrière^1*^, D. Daudé^2*^

^1^ Aix Marseille University, IRD, APHM, MEPHI, IHU-Méditerranée Infection, Marseille, France

^2^ Gene&GreenTK, 19-21 Boulevard Jean Moulin, 13005 Marseille, France

^3^ Service des Explorations Fonctionnelles Respiratoires Centre Hospitalo Universitaire Nord, Pôle Cardio-Vasculaire et thoracique, Assistance Publique des Hôpitaux de Marseille, Marseille, France

^*^Corresponding authors

Supplementary Figure 1 Growth of clinical isolates in presence (striped bar) or absence (empty bar) of phage cocktail. For each strain, bars represent the mean density (OD 600) after 16 h of incubation in MOPS glutamate with or without phage cocktail. Error bars represent the standard deviations of three replicated experiments. **p-values*<0.05; ***p-values*<0.01; ****p-values*<0.001 according to Student’s *t*-test.

Supplementary Figure 2 Dose response experiment of different concentration of *Sso*Pox-W263I on PA14 virulence against *A. polyphaga Linc AP1*. Curves represent the mean diameter of amoeba at different days of three experiments after incubation in the presence of bacteria treated with 10, 100 or 500 µg.ml^-1^ *Sso*Pox-W263I or with 500 µg.ml^-1^ *Sso*Pox inactive mutant 5A8. Error bars represent the standard deviations of three experiments.
